# Interprofessional Collaboration in the Mental Health Services in Norway

**DOI:** 10.1155/2014/849375

**Published:** 2014-03-02

**Authors:** Ellen Andvig, Jonn Syse, Elisabeth Severinsson

**Affiliations:** ^1^Faculty of Health Sciences, Buskerud University College, P.O. Box 7053, 3007 Drammen, Norway; ^2^Department of Health Promotion, Faculty of Health Sciences, Vestfold University College, P.O. Box 2243, 3101 Tønsberg, Norway; ^3^Centre for Women's, Family and Child Health, Faculty of Health Sciences, Vestfold University College, P.O. Box 2243, 3101 Tønsberg, Norway

## Abstract

The aim of this study was to describe and interpret interprofessional collaboration between healthcare professionals (HCPs) working at the district psychiatric centre (DPC) and employed in community mental health care (CMHC) using a dialogue-oriented co-operative approach. Data were collected by means of multistage focus groups and qualitative content analysis was performed. The main theme “development of interprofessional collaboration by means of organisational strategies and interactional styles” encompassed the following categories: “improved communication skills,” “developing structures for coordination and responsibility” and “ increased professional insight into the values and conditions necessary for decision-making.” In conclusion, more attention should be paid to leadership in terms of coordination and feedback. The HCPs must be acknowledged, understood and strengthened in their work to improve the quality of CMHC. Finally, we recommend that a range of organisational and administrative models of care be used in order to support improvement work.

## 1. Introduction

As in most other western countries, community mental health care (CMHC) in Norway has undergone many changes and a variety of care models and programmes have been introduced [1, 2]. The main objective of these reforms is to minimise institutionalisation by providing care and treatment within the context of the home, family, and immediate social environment [3]. In 2010, The Coordination Reform, a resolution for transforming the health care sector was passed by the Norwegian Government [1]. A major political reason was to ensure coordinated services for users.

The reform recommends strengthening service user participation in the development of all service levels as well as more systematic analysis and description of good patient pathways, which can promote improved coordination. However, persons with mental health problems (MHP) do not receive the help they want and require in a timely manner. Lack of coordination leads to comprehensive, long-term health problems, including mental illness and substance abuse [1]. Such patients are at high risk of experiencing adverse clinical events after discharge from hospital to their home [4]. Reform of the transition from institutional to CMHC requires collaboration between and coordination of CMHC and hospital care services.

### 1.1. Background

Interprofessional collaboration, a central model and ideal in Norwegian CMHC for decades, has recently been highlighted in a series of national health reforms and white papers. According to Orchard et al. [5, page 1], interdisciplinary collaborative practice means “a partnership between a team of health professionals and a client in a participatory, collaborative and coordinated approach to shared decision-making around health issues.” In the National action programme for mental health in Norway (Social and Health Department, 1999) [3], there is a clearly defined division of responsibility between municipal and state levels. However, the boundaries between these levels are blurred in everyday work with persons diagnosed with mental illness. As part of the specialist health care services, a District Psychiatric Centre (DPC) is expected to collaborate with the CMHC and provide out-patient services in the patients' community [6].

Availability, differentiation of treatment services, and collaboration were emphasised by Holst and Severinsson [7], who found a lack of continuity in the collaboration between CMHC and psychiatric hospital care. Different care ideologies and goals as well as inability to work together and make use of each other's competence rendered the collaboration between psychiatric care and community social services problematic [8].

Chong et al. [9] found that although healthcare providers acknowledged the importance of interprofessional collaboration, only a minority discussed it within the context of shared decision-making. When a collaborative network was established between in-patient staff, community staff, and discharged patients, the latter had a reduced rate of readmission and improved quality of life [10]. It is important to achieve a safe and seamless transition of patients from in-patient psychiatric units to CMHC [4]. Reynolds et al. [11] tested an intervention called transitional discharge, which included an overlap between in-patient and community staff, where the former continued to care for the discharged patient until a working relationship was established with a community care professional. Forchuk et al. [12] developed a transitional model that facilitated the discharge process by ensuring support from the hospital until a therapeutic relationship with a community care provider had been established.

Kozlowski and Ilgen [13] identified the following components and characteristics of well-functioning teams: common goals, performance of tasks relevant to goal achievement, ability to work independently, and undertaking different roles and responsibilities. In this way, the teams are able to combine their resources and coordinate knowledge, skills, and efforts to perform the necessary tasks. Prerequisites for such teams are trust, safety, and minimising and managing conflicts between team members. A well-functioning team thrives on information sharing, good communication, participative decision-making, and addressing new ideas, practices, or ways of organising treatment delivery [13]. Different care models are available. In Norway, the Chronic Care Model (CCM) has been implemented [14, 15]. The theory behind the CCM involves an innovative approach to user involvement and evidence-based guidelines. It consists of six components: community resources and policy; the health system and the organisation of health care; self-management support; delivery system design; decision support; and clinical information systems [16].

According to Reilly et al. [17], informational continuity and timely availability of information were poor in both community and specialist healthcare. It is essential that healthcare professionals (HCPs) move away from service-oriented delivery to a more person-centred and collaborative approach [18]. According to Borg et al. [18, page 86], person-centred care can be defined as “the provision of individualised health care that is closely congruent with and responsive to patients' wants, needs and preferences.”

The rationale behind the present study is threefold: (1) the difficulty of reorganising services in the light of the new reforms, (2) changes in professionals' responsibility for CMHC, and (3) models based on person-centred care.

### 1.2. Aim

The aim of this study was to describe and interpret interprofessional collaboration between healthcare professionals (HCPs) working at the district psychiatric centre (DPC) and employed in community mental health care CMHC using a dialogue-oriented cooperative approach.

## 2. Methods

### 2.1. Design

An action research approach was applied [19]. Action research encompasses a range of methods where the main intention is to contribute both practical and conceptual knowledge by conducting research with rather than on people [20]. Due to its dialogue-oriented, cooperative approach, action research has the potential to facilitate understanding of and lead to changes in the field [21]. Cooperative research can therefore play an important role in assisting HCPs to integrate theory and research in the clinical setting, as it includes not only practical aspects but also the knowledge or theory on which the actions are based [21]. The present research investigates the collaboration between DPC and CMHC professionals in one community and a University College in Norway.

### 2.2. Recruitment and Participants

Participants from the DPC were self-recruited [22]. All professionals in the DPC unit were invited to participate in the initial phase of the project and those who were interested could take part in the follow-up multistage focus groups. The inclusion criterion for the participants from CMHC was working with people with MHP who sometimes required care at the DPC. A total of 18 professionals participated, ten from the DPC and eight from the CMHC. Continuity was ensured by the fact that half of the participants attended all sessions. The participants from the DPC ranged in age from 30 to 60 years and comprised psychiatric nurses (4), assistant nurses (2), social educators (2), a social worker (1), and an occupational therapist (1). The participants from the community ranged in age from 40 to 55 years and were registered psychiatric nurses (7) in addition to a social educator (1).

### 2.3. Dialogue-Based Discussions

In cooperative research, which includes dialogue-based discussions characterised by an interchange of clinical experience and theoretical reflections, the focus is on developing knowledge for action [21]. The dialogue-based discussions in the present study pertained to collaboration in the area of person-centred care [23] and were intended to facilitate the articulation of practical and tacit knowledge. In-depth reflection was promoted and stimulated by the expression of different experiences in the group [24]. The following aspects were addressed: person-centred care; user participation in theory and practice; collaboration with relatives; collaboration between DPC and CMHC as well as the roles and responsibilities involved. The dialogue-based discussions took place at the DPC and comprised six sessions, each of which lasted for approximately one hour.

### 2.4. Data Collection

Data were collected by means of six multistage focus group interviews where aspects of interprofessional collaboration were addressed [24]. Focus groups are a data collection method that offers several advantages when the participants constitute a homogeneous group [25]. Multistage focus groups imply that the knowledge shared is enhanced over the course of several meetings, thus leading to a deeper understanding of the agreed topic of interest [24]. The participants from the two workplaces each had two focus group sessions, after which two interviews took place at which both groups were present. The focus group interviews were conducted by the first (E.A.) and second authors (J.S.), who took turns to act as moderator and lead the discussion and as comoderator who documented the sessions by taking notes and summarising the discussion. Each interview lasted for about 90 minutes, was audio-taped, and subsequently transcribed verbatim. All interviews were held at the DPC. Examples of guiding questions were: in what way do you collaborate to provide patient oriented care? and how do you collaborate with relatives?

### 2.5. Data Analysis

Qualitative content analysis was used to analyse the data [26] in a stepwise manner. The transcribed text of the first focus group interview was condensed and an initial analysis performed before the next meeting. The second interview focused on topics that arose from the first meeting. Finally, the main theme, categories, subcategories, and codes generated in the analysis of the data from all six focus group sessions were identified, named, and summarised (Table 1). Statements from the interviews were systematised by grouping them under different codes. The content of the categories was clarified, checked against the transcribed interviews, and validated by statements from the interview texts. Coherent majority and minority perceptions were searched for and illuminated by abstraction in addition to specific examples related to the themes [26]. A preliminary analysis was presented to two of the coresearchers, after which it was discussed and revised.

### 2.6. Ethical Considerations

Approval for the study was granted by the Regional Ethics Committee of South Norway (no. S-09188b) and from the human resource managers of the municipality and the DPC in question. In addition, the principles of confidentiality, voluntary participation, and informed consent were applied [27]. No information that could identify the participants was included in the final report.

## 3. Results

The main theme “development of interprofessional collaboration by means of organisational strategies and interactional styles” encompassed the following categories: “improved communication skills,” “developing structures for coordination and responsibility,” and “increased professional insight into the values and conditions necessary for decision-making.”

The main theme included the following elements: managing, connecting, acknowledging, and confirming. The evidence of the development of a new system to enable collaboration on various organisational levels revealed the following: (a) the need for common guidelines for innovative and person-centred approaches to care and (b) the necessity of ensuring safe and secure care by means of support, data collection, documentation, and improved access. The evidence of development on an interactional level comprised the following: accepting the challenges inherent in clinical practice; experiencing that interaction leads to shared understanding; sharing experiential knowledge strengthens the substance of communication; and the ability to discuss the care pathway from institution to community.

### 3.1. Improved Communication Skills

This category contained the subcategories “getting to know each other” and “development of a common professional understanding.”

#### 3.1.1. Getting to Know Each Other

Both groups of staff (hereafter referred to as teams) emphasised the importance of meeting face to face to become familiar with and understand each other. Such meetings made it easier to contact and communicate with each other and led to more flexible collaboration.

*When you see a face and speak to her/him, you are immediately connected and understand the situation sooner compared with what happens when you have never spoken in person.*

The members of both teams emphasised that to understand and know each other involved clarifying each other's expectations. They discussed several examples of lack of collaboration caused by differing expectations of the other's contribution to patient care.

#### 3.1.2. Development of a Common Professional Understanding

The teams emphasised the importance of having a common professional understanding of the goal of patient care. They recognised the need to be familiar with each other's way of working, different skills, and professional competencies. They highlighted the necessity of discussing a variety of topics, including their understanding of terms such as “rehabilitation” and “recovery process.” They considered it necessary to focus on the meaning of these terms in practice, both in relation to in-patients and out-patients. They acknowledged that they had different understandings of a patient's overall MHP and recovery process. They therefore wished for a general discussion during which they could raise questions related to patient care, the medical illness model dominant in DPC, and the person-centred model in CMHC. The following is an example of an issue that arose.

*Should a patient stay in an institution until she/he has recovered from her/his symptoms, or is it better to support her/him to cope with everyday life in her/his own home?*

### 3.2. Developing Structures for Coordination and Responsibility

This category included two subcategories: “routines” and “regular meetings.”

#### 3.2.1. Routines

The teams experienced a lack of routines for information exchange. The DPC staff usually provided information to CMHC staff by phone, but the latter reported that the information never reached them. Documentation of milieu therapy from the DPC to the CMHC was sparse and insufficient. One DPC staff member related.

*We usually send information about an epicrisis to the patient's physician, but we often forget to send a summary of what we have done in milieu therapy.*

DPC staff lacked information about the kind of MHC the patient had received before she/he was sent to the DPC, especially in cases of a first admission. The teams wanted clear routines for information exchange about patients' medication. The DPC and CMHC teams agreed on the exchange of written information about the patient when she/he is to be transferred from one to the other.

#### 3.2.2. Regular Meetings

The teams agreed that an important aspect of fruitful collaboration is the establishment of regular meetings for coordinating patient care. When the patient has a responsibility group, which works as a team together with her/him (cf. [28]), group meetings are the most important tool for coordination. The group formulates a plan that states the patient's own goals and the professionals' responsibility for care and support, for example, how the patient can obtain assistance should her/his problems become worse. This overall plan was used for the patient by the DPC and CHMC teams, both of which already had positive experiences of such groups.

*Where we have established a responsibility group around the patient it is easier to collaborate. We gain a shared understanding of the work. It is difficult but worthwhile.*

The teams also agreed that responsibility group meetings should be better organised and the participants made accountable for being present at meetings, which should be prepared in good time and put on a schedule.

If the patient did not have a responsibility group, the teams agreed to hold meetings to discuss collaboration in advance of the patient's admission to the DPC, and before her/his discharge from the DPC to CMHC.

The patient's primary contact from the DPC and one CMHC professional, who knows the patient, should be present at such meetings in order to clarify the patient's expectations, formulate plans for future care, and enable information exchange between the DPC and CMHC on the actual situation and follow-up. It was also agreed to hold monthly lunch meetings to discuss how to maintain the quality of the patient's care.

### 3.3. Increased Professional Insight into the Values and Conditions Necessary for Decision-Making

This category comprised the following subcategories: “increased user involvement,” “interactional flexibility in decision-making,” and “equality and respect.”

#### 3.3.1. Increased User Involvement

The team members agreed that the patient had to be more involved in the treatment and decisions concerning her/his own person and that user collaboration also meant the involvement of relatives. The focus groups enabled the professionals to become more aware of the patient's wishes, care needs, and hopes for the future, which facilitated them to find better solutions to her/his problems.

*It's important that the patient participates in the dialogue about her/his own life and expresses her/his concerns about the treatment, care and future needs. If the patient does not do so, she/he will feel like a pawn on a chess board.*

#### 3.3.2. Interactional Flexibility in Decision-Making

This subcategory focused on flexibility in decision-making pertaining to admission, discharge, and overlap between the two organisations. One example was planned admissions. Patients could be admitted to the DPC for a short period (one to two weeks) in accordance with their needs and in agreement between the patient, DPC, and CMHC to prevent deterioration in the patient's condition.

*We frequently gain a mutual understanding of the purpose of the admission by means of discussions between the DPC, MHC and the patient.*

From the HCP point of view, planned admissions were a preventive strategy to avoid compulsory admission and treatment. According to the informants, such admissions were socially and economically beneficial. Another example was the use of flexible discharge from the DPC to CMHC, which was carried out gradually by allowing the patient to live at home but keeping her/his hospital bed available. The DPC staff members followed up the patient in her/his home and were formally responsible for the care. Depending on the patient's needs, MHC personnel could also be involved in her/his care. In the focus groups, the CMHC personnel shared their experiences by means of a metaphor.

*We put on our “outdoor shoes” and visit the patient at home. Then we understand far more of the patient's life situation and the methods used by the DPC.*

#### 3.3.3. Equality and Respect

The participants reported that in several cases collaboration resulted in improved patient care and a better service.

*We respect our different contributions. We complement each other in patient-related duties because we have different competencies. *

However, in other cases the CMHC staff claimed that they were not given credit for their professional work and that it was not considered as important as institutional psychiatric treatment.

After the focus group discussions the two teams stated that they understood more about their similarities and differences. They agreed on the necessity of acknowledging the equal value of their contributions. Equality meant collaboration based on mutual respect and recognition of each other's roles, values, and competencies.

## 4. Discussion

The aim of this study was to describe and interpret interprofessional collaboration between DPC and CHMC by means of a dialogue-oriented cooperative approach. The main theme “development of interprofessional collaboration by means of organisational strategies and interactional styles” encompassed the categories: “improved communication skills,” “developing structures for coordination and responsibility,” and “increased professional insight into the values and conditions necessary for decision-making.”

According to the findings, the HCPs improved their *communication skills* and developed some common knowledge due to reflection on practice. Getting to know each other was a prerequisite for this development [13]. The teams learnt different perspectives on person-centred care and collaborative practices from each other and discussed their different professional skills. The findings provided evidence that the HCPs prepared the ground for a common understanding among themselves as well as with regard to patient care. However, their dialogues also revealed and made them more aware of the differences between them. Meeting at a monthly forum provided an opportunity to facilitate a *common understanding* of their different skills, which is supported by [7]. The two teams developed a greater awareness of different types of knowledge, such as their own experiential knowledge and skills. They discovered the importance of acting upon research evidence as well as the patient's knowledge and experience in relation to decision-making. These findings are supported by Rolfe et al. [29], who reported that nurses mostly draw on their own experiences when making decisions and that research has relatively little impact on their practice. Thus, highlighting different types of knowledge provides healthcare staff with a repertoire of alternative actions.

*Developing structures for coordination and responsibility* indicated a need for *routines* such as *regular meetings as well as distinct leadership.* It was vital for teams to develop routines for exchange of information, as pointed out by Kvarnström [30]. Magnusson and Lütz$\overset{´}{\text{e}}$n [8] reported that lack of information between teams can result in low satisfaction on the part of team members, other professionals, and patients. The findings indicate the absence of accountability as well as the importance of achieving consensus at system level. This is in accordance with Holm and Severinsson [31, 32] who stress that the leader's duty is to increase collaboration between the discharging unit and the community health care services, in addition to clarifying role expectations and areas in need of development. In this process, an important task for the leader is to make the personnel responsible for following the agreed task descriptions, frameworks, and quality systems.

The findings revealed that the teams had a great deal of good intentions, will, and interest in collaboration. They presented several examples of how they collaborated in individual cases and pointed out the need for more systematic collaboration. This is in accordance with Holst and Severinsson [7], who found that collaboration between the municipality and hospital took the form of ad hoc meetings. The respondents underlined the need for more structured professional guidelines and collaboration in order to improve the quality of CMHC. According to Petri [33], it is essential that the individuals in the collaboration process are committed to the decisions made and assume responsibility for adhering to them. Petri emphasised that the persons collaborating must support each other and believe that collaboration leads to quality care. This is especially important when it comes to the primary contacts in this study. The team members should understand their duty to participate in responsibility groups and other relevant meetings agreed upon. Formal interprofessional collaboration procedures consist of regular interprofessional meetings, such as those of responsibility groups. The main goal of the responsibility group is to share responsibility, exchange information, and ensure coordination and progress [28]. 

*Increased Professional Insight into the Values and Conditions Necessary for Decision-Making.* The findings revealed two different perspectives on care: the medical illness model and the person-centred model. Lack of shared understanding with the patient can be a consequence of a mental health system based primarily on the medical illness model. The DPC professionals usually withheld information about the treatment plan and medication, thus effectively maintaining control and power over the patient. According to Chong et al. [9], HCPs appeared to have differing perceptions of the appropriate level of user involvement in shared decision-making. It is important to allow patient preferences to influence practice to a greater extent (cf. [29]). The findings indicate that the DPC professionals developed new knowledge about decision-making, including patient involvement as well as flexible solutions based on the patient's opinion of what is best for her/him. These findings are in accordance with the shared decision-making systems in person-centred care described by McCormack and McCance [23]. An additional finding was that both the DPC and the CMHC personnel developed a more holistic approach to patient care, viewing the patient together with her/his family and local community. The professionals understood more about the importance of creating smooth pathways across organisations, as described by Reynolds et al. [11]. Smooth pathways included ensuring patient support from the hospital until relationships with community care had been established (cf. [12]). Open communication with patients and families as well as collaboration among healthcare providers can ensure a seamless transition to the next care level and facilitate best patient outcomes [4].

Furthermore, this study indicates the need for mutual *trust and respect* when determining the professionals' collaborative roles. The issue of inequality between the DPC and CMHC was stressed. According to Holm and Severinsson [34], creating an environment characterised by trust and mutual respect is important. Distrust can have serious implications for discharge planning collaboration and be an obstacle to reciprocity and equality. Reciprocity can be understood as an ongoing process of exchange with the aim of establishing and maintaining equality between parties [35]. Trust is a central aspect of collaboration and concerns trusting that others manage their work and have good intentions [36]. Personal contact by means of regular meetings can lead to trust between the teams, which according to Magnusson and Lütz$\overset{´}{\text{e}}$n [8] is an important prerequisite for and enhances collaboration. The findings revealed that the two teams previously viewed each other as “us” and “them.” It was essential that the two teams started to see each other as colleagues.

In order to address the power relationship it is important for both the DPC and the CMHC team to have a mutual commitment to collaboration by fostering a group dynamic in a noncompetitive atmosphere. Otherwise, power based on the dominant care model will be favoured, resulting in minority voices being silenced [35]. The insights into their different as well as common perspectives gained by the participants in this study provided them with valuable cultural information including ideas for sharing information about their thinking and work practices. Reciprocity was evident at both individual and system levels.

### 4.1. Methodological Considerations

The research model used in this study was characterised by cooperation and flexibility. Knowledge was developed in dialogue-based discussions between the professionals involved, rather than transfer from informants to researchers. The study sample represented the staff categories in the DPC and CMHC and the 18 participants provided a broad range of opinions. They played an active part in the focus group interviews and learned from each other's experiences. A strength of the multistage focus group method was that the experiences shared between the participants could be the object of reflection, development, and a deeper understanding (cf. [24]). The presentation of a preliminary analysis to the participants before each focus group interview contributed to trustworthiness [26]. In the final analysis, transparency and rigour were ensured by adhering to prescribed data analysis steps and by the fact that the text was analysed by the three authors. One limitation is that the sample was drawn from a single health district.

## 5. Conclusion

In conclusion, interprofessional collaboration based on communication, shared decision-making, and knowledge of professional responsibility can enhance the quality of care. In addition, more attention should be paid to leadership in terms of coordination and provision of feedback. HCPs need to be acknowledged, understood, and strengthened in their work to improve the quality of CMHC. Finally, we recommend that a range of organisational and administrative models of care be used in order to support improvement work.

## Figures and Tables

**Table 1:** Summary of the findings: main theme, categories, subcategories, and codes.

Main theme: development of interprofessional collaboration by means of organisational strategies and interactional styles		
Categories	Sub categories	Codes
Improved communication skills	Getting to know each other	(i) Meeting and getting to know each other face to face (ii) Clarifying expectations
	Development of a common professional understanding	(i) Recognising each other's professional reasoning and tasks (ii) Joint professional discussions on the understanding of high quality patient care

Developing structures for coordination and responsibility	Routines	(i) Lack of routines for exchange of information (ii) Written information should be routine (iii) Joint meetings with the patient to clarify her/his needs and agree on responsibilities
	Regular meetings	Responsibility groups are important for coordinating patient work

Increased professional insight into the values and conditions necessary for decision-making	Increased user involvement	(i) We need greater awareness of how to involve the patient (ii) Dialogue with the patient about her/his views of the follow-up and her/his care needs (iii) Collaborate with the patient
	Interactional flexibility in decision*-*making	(i) We have positive experiences of being flexible when working with planned admissions (ii) Very positive for the patient when we (DPC and CMHC) overlap at discharge
	Equality and respect	(i) A top-down attitude inhibits collaboration (ii) Sometimes we experience a “little sister-big brother” attitude from DPC staff (iii) We must respect each other's contributions

DPC: district psychiatric centre; CMHC: community mental health care.
